# Effect of public health expenditure on maternal mortality ratio in the West African Economic and Monetary Union

**DOI:** 10.1186/s12905-024-02950-2

**Published:** 2024-02-09

**Authors:** Jacques Boundioa, Noël Thiombiano

**Affiliations:** Thomas SANKARA University, Department of Economics, Economic and Social Studies, Documentation and Research Center (CEDRES), Ouagadougou, Burkina Faso

**Keywords:** Public health expenditure, Maternal mortality, Two-step least squares

## Abstract

**Introduction:**

Maternal mortality in West African Economic and Monetary Union countries (WAEMU) is the highest compared with other regions in the world. The majority of health care sources in WAEMU are private and largely involve out-of-pocket expenditures, which may prevent healthcare access. Maternal mortality is an important indicator of the level of attention given to mothers before, during and after childbirth and thus of a system’s overall coherence and capacity for anticipation. Therefore, the objective of this study is to analyze the effects of public health expenditure on maternal mortality in WAEMU.

**Methods:**

The study used panel data from the World Bank Development Indicators (WDI) from 1996 to 2018 covering 7 countries in the West African Economic and Monetary Union. The two-step least squares (2SLS) on health demand function was used to test the effect of public health expenditure on maternal mortality.

**Results:**

Public health care spending showed a significant negative association with maternal mortality. However, private health expenditure was positively associated with maternal mortality.

**Conclusion:**

Public health care expenditure remains a crucial component of reducing maternal mortality. In this region, the authorities need to increase public health spending to build more health centers and improve the equipment of existing infrastructures. Additionally, it is important to reduce the financial barriers for pregnant women. To this end, the operationalization of universal health insurance could help reduce these financial barriers by reducing direct household payments.

## Introduction

Health is “a state of complete physical, mental and social well-being and not merely the absence of disease or infirmity“ [1]. It is a durable capital stock that produces healthy times for market and non-market activities [2]. The health status of a country depends on maternal and infant mortality rates, which play a critical role in assessing population health, health system quality, and socioeconomic status [3]. Maternal mortality, defined as “the death of a woman during pregnancy or within 42 days of termination of pregnancy,” but excluding deaths due to accidents/incidents [4], plays a role in socioeconomic development. Maternal health is likely to bring economic and non-economic benefits to households and thus to society. It creates opportunities for job creation, improves people’s health, provides social and economic stability, and thus leads to development. A healthy mother gives birth to a healthy child, while a mother with poor health may give birth to a child with poor health, which may affect the quality and quantity of the labor force in the long run.

In addition, women make up a significant proportion of the working population in developing countries, which are predominantly rural and agricultural. Consequently, maternal deaths could lead to a reduction in both the quantity and quality of the labor force, which would have a negative impact on economic growth.

However, the maternal mortality ratio remains a worrisome phenomenon in many developing countries, despite declines in recent years. In fact, approximately 810 women die during pregnancy each year, and almost 94% of them are in developing countries [5]. Although this rate decreased by 38% between 2000 and 2017, from 342 to 211 per 100,000 live births, 295,000 women died worldwide in 2017 as a result of pregnancy and childbirth. We note that it is highest in developing countries and especially in sub-Saharan Africa (SSA), with 534 deaths per 100,000 live births and 200,000 maternal deaths (i.e., approximately 68% of total deaths worldwide) in 2017. Nevertheless, the maternal mortality rate also declined in SSA, falling from 870 to 534 between 2000 and 2017, a decrease of approximately 38%.

Like other regional groupings, the West African Economic and Monetary Union (WAEMU) has seen a decline in its maternal mortality rate. Between 2000 and 2017, it fell from 705 to 473 maternal deaths per 100,000 live births, a 33% decrease. However, compared to other regions, WAEMU had the highest number of maternal deaths, but the decrease was also small compared to SSA. In 2017, the global average, North Africa and Latin America and the Caribbean were 211, 112 and 73 mothers per 100,000 live births, respectively, compared to 473 in the WAEMU.

Given the importance of maternal health to economic development and the high number of maternal deaths, the WHO emphasizes the need for health systems to reduce preventable mortality through early intervention. For this reason, the Sustainable Development Goals (SDGs) for 2016–2030 aim to reduce the maternal mortality ratio from 315 in 2015 to below 70 in 2030 (Goal 3). The Millennium Development Goals (2000–2015), in their Goal 5, aimed to improve maternal health by reducing the maternal mortality ratio by ¾ or 263 per 100,000 live births.

The West African Health Organization (WAHO) strategic plan for 2016–2020 also includes maternal health promotion as priority 1. In addition, the first Economic Community of West African States (ECOWAS) Forum on Best Practices in Health was held in July 2015 in Ouagadougou, focusing on “Ending Preventable Maternal and Child Deaths in West Africa.”

In many countries, public health expenditure is the best public policy tool for improving health indicators. Public health expenditures refer to all operating and capital expenditures from the state budget, external resources channeled through the government and social security. They enable investment but also ensure the functioning of the health sector.

Empirically, debates on the relationship between public health expenditure and health indicators are ambiguous [6]. There is some controversy about the effect of public health spending on improving population health. Some authors [7–9] indeed find a positive effect of public health expenditure on health. Others [10, 11], however, find a non-significant effect or a negative and significant effect of public health spending on population health.

Given the evidence, there are inconsistencies regarding the effect of public health spending on improving population health. Maternal mortality rates in the WAEMU remain high compared to the world average. Moreover, despite the observed decline, it would be difficult for WAEMU countries to achieve Goal 3 of the SDGs by 2030.

Furthermore, to our knowledge, the direct effect of public health expenditure on maternal mortality rate has not been studied in the WAEMU. The only studies we know of in the WAEMU region are those by [8, 12]. They analyzed, respectively, the macroeconomic determinants of the infant mortality rate and the determinants of health status in the region, which is approximated by the infant and child mortality rate.

However, maternal mortality is also an important indicator of the degree of attention given to mothers before, during, and after childbirth and thus of the overall coherence and anticipatory capacity of a system. Improving maternal health reduces infant and child mortality rates. In fact, motherless children are ten times more likely to die prematurely than other children [13].

The additional contribution of this article concerns methodological aspects. Most studies have not considered the problem of bidirectional causality between public health expenditures and health indicators in their estimations. They have used OLS as estimators, so bias in their estimates is to be suspected.

The aim of this article is to analyze the direct effect of public health expenditure on the maternal mortality rate in the WAEMU.

The remainder of the article is organized as follows: The first section addresses the empirical discussions, and the second section is devoted to the methodology and the discussion of the estimation results. A final section is devoted to the conclusion and economic policy implications.

### Theoretical framework of public health expenditure and maternal mortality

Two main theories are used in this article: the health capital theory of [2] and the law of [14]. Grossman’s theory essentially analyses the way in which individuals allocate their resources to produce health. It is based on constrained utility maximization, in which an individual seeks to maximize his utility with a given set of resources. It postulates that individuals seek to maximize their health by investing in themselves in order to produce the desired typical state of health. Thus, this theory presents the individual as a person whose demand for health inputs is a derived demand, not necessarily for consumption, but to produce a typical health outcome. To do this, the individual acquires health inputs such as health care financing, whether public or private, education, environment and income in order to engage in a production process that results in a typical health outcome. In essence, this theory explains much of the relationship between health financing and health outcomes.

Regarding [14], while arguing in favor of public spending, asserted that there are inherent trends towards increased public spending on investment. For example, the increase in public spending is explained by the government’s need to provide social services such as public health through budgetary expenditure.

Based on these theories, maternal health through the maternal mortality ratio could be influenced by public spending on health but also by other variables such as education, private spending, etc.

### Empirical discussions on the effect of public health spending on population health

There is empirical literature on the effect of public health spending on health indicators at the level of both developed and developing countries and sub-Saharan Africa [9, 15, 16]. However, the results found by the authors are controversial. For example, some authors find that public spending positively affects health outcomes in different countries [9, 17]. Others show that such spending has no effect or a negative effect on health indicators [11, 18].

### Positive effect of public health expenditure on health outcomes

Various health indicators have been used in the literature to analyze the relationship between public health expenditures and population health. These indicators include infant, child, maternal, and neonatal mortality rates and life expectancy at birth [12]. examined the determinants of health status in WAEMU countries. Using an econometric approach based on the least squares estimator corrected for dummy variables (LSDVC) for the period 2000–2014, they found that public health expenditure is an important determinant of health status. The limitation of their study lies in the method used. While the LSDVC method corrects for endogeneity due to measurement error, it does not account for bidirectional causality. The health status of a population can justify the level of public health spending and vice versa [8]. , using the double least squares fixed effect method, also finds that, in addition to female literacy, GDP, and urbanization, public health spending as a percentage of GDP has a positive effect on the infant mortality rate.

Along these lines [17], analyzed the relationship between health care financing and health indicators for 10 oil-producing countries in Africa. Specifically, they examined the effect of public health spending, private health spending, and external resources allocated to health on four health indicators: maternal, infant, and child mortality rates and life expectancy at birth. Using a fixed and random effects model, they found that these different funding sources improved health indicators in these countries. This study did not consider the problem of endogeneity that might exist in the relationship between health expenditures and indicators. Studies have been conducted with other data sets that use panel data. For example, [19] examined the impact of health spending on infant and neonatal mortality rates in 46 SSA countries. They used World Bank data for the period 2000–2015 and, using the fixed-effect method, found that public spending and foreign aid had a significant effect on health. Private health spending, however, has no significant effect on infant mortality rates [6, 7, 20]. found similar results in SSA and the 25 Nigerian states [21, 22]. also found that public health spending reduces infant and child mortality rates.

Using time series data, [23] analyzed the effect of public health spending on health outcomes over the period 1980–2018 in Nigeria, using the maternal mortality ratio as a proxy. Using a long-term autoregressive model, they conclude that public health spending leads to a long-term decline in the maternal mortality rate. This study was conducted in a country whose characteristics (standard of living and population) differ somewhat from those of WAEMU, making generalization of the results impossible. An earlier study by [24] on Nigeria, using the Granger estimation method, reached the same conclusion that public health spending reduces the child mortality rate. Similar results have been found in other countries, such as Ghana and Algeria [25, 26].

In addition to mortality rates (infant, neonatal, child, and maternal), other authors have included life expectancy at birth in their analysis. For example, [27] examined the impact of health expenditures on health indicators in OECD countries. They used life expectancy at birth for men and women and maternal and infant mortality rates. They conclude that public health spending leads to an increase in life expectancy at birth but a decrease in mortality rates [28, 29]. reached a similar conclusion using infant, child, and neonatal mortality rates and life expectancy at birth in selected East African countries and Nigeria, respectively [30]. also reached this conclusion using life expectancy at birth, infant and child mortality rates, and neonatal mortality rates.

### Negative or no significant effect of public health expenditure on health indicators

Many empirical studies [18, 26] have found that public spending on health leads to deterioration in health indicators or has no effect on health indicators.

Indeed, [31] examined the effect of public health spending on health indicators in India. Using the OLS method, they conclude that public, total, and private health spending have no effect on infant or child-maternal mortality rates or life expectancy at birth. Inefficiency in public health spending could be the main reason [26]. find a similar result in Algeria using an ARDL cointegration approach. They explain this result by noting that life expectancy is a quantitative indicator and that the use of health and long-term care services does not necessarily increase this indicator but rather improves the well-being of the individual [32]. also studied the effects of public and private health spending on health indicators in 40 SSA countries over the period 2000–2010. Using a fixed effects OLS method, they conclude that public health spending has a negative sign but does not significantly affect maternal mortality [10, 33] and [34]. have previously found similar results. According to these authors, the problem lies in the design of public policies to reduce excess mortality and morbidity in developing countries [35–37]. have also shown that public spending on health care is not a determinant of population health status in these countries. They explain this by the poor design of public health policies.

 [15] analyzes the determinants of health in developing countries. Using the GMM method, he also concludes that government health spending has no effect on reducing mortality in developing countries. Inefficient provision of health services and initial poor health status could be the main reasons. Indeed, countries with very low health indicators would need to allocate a great deal of resources to have a significant effect [11, 38]. , using life expectancy at birth as an indicator, find that public health spending has no effect in 29 OECD countries and in the Eastern Mediterranean regions, respectively. The quadratic effect of public health spending was considered by [39]. Using the infant survival rate in Togo and an error correction model from [40], he shows that public health spending has no significant effect on health outcomes below a certain threshold.

Other authors conclude in their study that public health spending contributes to the deterioration of the health status of the population. Indeed, [18] examined the relationship between health inputs, health outcomes, and public health spending in BRICS countries. Based on panel data and using the OLS method, he finds a positive elasticity between public health spending and mortality rates. This means that an increase in public health spending leads to an increase in mortality rates or a deterioration in health indicators in these countries. This is because the marginal benefit of increasing public health spending may be small compared to the marginal cost of high taxes if the financing comes from taxes. These taxes therefore affect preventive health spending [41]. also examined the effects of public financing of health expenditures, health insurance, and other factors on health production using data from 20 OECD countries over the period 1960–1992. They found that mortality rates depended simultaneously on health spending and the choice of health insurance system. Specifically, an increase in the proportion of public funds devoted to health spending led to an increase in mortality rates. According to the authors, this result may be due to low efficiency in health care delivery or to different combinations of public financing. Governments need to consider efficiency when financing health care.

The critical analysis of these results lies in the use of health indicators, which differ from study to study, and in the econometric methods used. Most authors used indicators such as infant and child mortality rates and life expectancy at birth. The indicator of maternal mortality is poorly considered in these studies, especially in developing countries and particularly in WAEMU countries.

This research covers a wider time period and relatively recent data compared to [32] in the case of Sub-Saharan Africa. Second, in their study as many authors, both fixed and random effects estimation methods were used, which could bias the results. Thus, by using a more robust empirical strategy, such as the double least squares (2SLS) method to account for the potential endogeneity problem, this research offers new empirical evidence regarding health expenditure (public and private) and its effect on maternal mortality in a panel setting.

In addition, it is important to note that all WAEMU countries have implemented maternal health policies but still have the highest mortality rates. However, previous studies in this area have focused on time series data with some countries taken in isolation and do not cover a long period. This research uses a relatively long period, which makes it possible to take into account the wide-ranging healthcare reforms that have been carried out over the years in this area.

Most previous studies have ignored some factors such as population density, the incidence of HIV/AIDS and the inclusion of private expenditure in their analysis.

Finally, the effect of public spending on health depends on the methods used and the control variables included in the model [42]. The present analysis aims to overcome the limitations of these previous studies by focusing on the case of the WAEMU region and using the maternal mortality ratio as an indicator.

## Method

### Data and variables

This article focuses on seven (07) WAEMU countries and covers the period 1996–2018, where data are available. The data used are from the [43].

The maternal mortality ratio is used in this article. It is defined as the number of women who die per 100,000 live births. This variable is one of the main indicators used in the literature to approximate health production [4, 20]. In addition, the various international and subregional development goals and national health development plans have a major impact on these indicators, especially maternal mortality, in terms of health. An increase in public resources allocated to health leads to better access to health services for pregnant women. In the literature, authors have shown that there is a negative relationship between public health spending and maternal mortality rates [20]. In this study, public expenditure includes domestic revenue as internal transfers and grants, transfers, subsidies to voluntary health insurance beneficiaries, nonprofit institutions serving households or enterprise financing schemes, compulsory prepayment and social health insurance contributions. We postulate a negative relationship between public health expenditure as a percentage of GDP and maternal mortality rate. Gross domestic product (GDP), used as a proxy for national income, can affect mortality rates in direct or indirect ways [44]. First, there is a direct and positive relationship between wealth and survival, which depends on the ability of households to secure the supply of goods and the demand for medical products. An increase in income leads to an increase in demand for healthcare. This is because, according to economic theory, if health is considered a necessary good, an increase in income, other things being equal, leads to an increase in the demand for health care services.

At the macroeconomic level, [45] explained that population health improves as per capita income increases. As income increases, the government could increase the supply of health services to the population. This suggests that higher income is associated with lower maternal mortality rates. Education in general and that of women in particular is an important determinant of household health, especially in relation to prenatal and postnatal care in developing countries [42, 46]. have shown that women’s educational attainment plays a critical role in the health status of infants and children, as well as the population as a whole. An additional year of education per woman reduces infant and maternal mortality rates. Finally, [47] show that female education has a positive effect on reducing infant mortality. However, given data availability, we approximate this variable by secondary school enrollment rates. Therefore, a negative sign is expected for the maternal mortality rate. The incidence of HIV/AIDS was defined as the number of new cases in a given year. AIDS may lead to an increase in the maternal mortality rate. Indeed, AIDS and complications during pregnancy and childbirth remain the leading causes of death among women of childbearing age. HIV infection is now the leading cause of death among adults aged 15–59 years, nearly 3 million people died from AIDS in 2006, 80% of whom died in SSA. In their study, [29] state that AIDS negatively affects the health of the population in Nigeria. This variable is believed to have a positive sign on maternal mortality rate.

The literature uses private health spending (as a % of GDP). Private expenditure includes funds from households, corporations and nonprofit organizations [48]. have shown that an increase in private health expenditure leads to an increase in life expectancy at birth and a decrease in infant mortality rate. However, in low-income countries in general and WAEMU countries in particular, a large proportion of the population is affected by poverty. Therefore, an increase in private health spending may limit access to health care. The expected sign of private health expenditure is positive.

Population density, defined as the ratio of a country’s population to its total land area in square kilometers (including uninhabited areas), according to the [43], affects the health of populations. Since 1961, WAEMU countries have experienced high population concentrations. For example, the population density in Burkina Faso increased from 37.91 persons/km2 in 1996 to 72.19 persons/km2 in 2018 ([43]. This population concentration affects investment in health care and, in turn, health. When populations are concentrated, it encourages public authorities to build social infrastructure. This, in turn, facilitates access to health care because people must travel fewer distances [49]. show that population density contributes to better health not only by reducing the per capita cost of health services but also by reducing the distances people must travel. However, population density can also adversely affect people’s access to health services. The denser the population, the more difficult access to health care can become as demand increases and per capita consumption of health care services decreases. Since health care is a typical example of an impure public good, concentration can lead to congestion. In addition, density leads to environmental degradation, which negatively affects people’s health [50]. An indeterminate sign is expected for this variable.

### Model

The specification is based on [2] theoretical microeconomic health production function model, adapted to the macroeconomic level by [51].

The empirical specification can be presented as follows:

$$\eqalign{ {\varvec{l}\varvec{n}\varvec{M}\varvec{M}\varvec{R}}_{\varvec{i}\varvec{t}} & ={\varvec{\alpha }}_{\varvec{i}}+{\varvec{\beta }}_{1}{\varvec{P}\varvec{H}\varvec{E}}_{\varvec{i}\varvec{t}}+{\varvec{\beta }}_{2}\varvec{l}\varvec{n}{\varvec{G}\varvec{D}\varvec{P}}_{\varvec{i}\varvec{t}}+{\varvec{\beta }}_{3}{\varvec{P}\varvec{r}\varvec{H}\varvec{E}}_{\varvec{i}\varvec{t}}\cr & +{\varvec{\beta }}_{4}{\varvec{P}\varvec{D}\varvec{s}}_{\varvec{i}\varvec{t}}+{\varvec{\beta }}_{5}{\varvec{I}\varvec{N}\varvec{C}\varvec{V}\varvec{I}\varvec{H}}_{\varvec{i}\varvec{t}}+{\varvec{\beta }}_{6}{\varvec{S}\varvec{S}\varvec{E}}_{\varvec{i}\varvec{t}}+{\varvec{\epsilon }}_{\varvec{i}\varvec{t}}}$$

${MMR}_{it}$ represents the maternal mortality ratio of country i at period t; ${PHE}_{it}$ represents public spending on health as a percentage of GDP for country i at period t; ${GDP}_{it}$ is the gross domestic product per capita in constant for country i at date t; ${PrHE}_{it}$ is private health expenditure; ${PDs}_{it}$ represents population density; ${INCVIH}_{it}$ is the incidence of HIV/AIDS; ${SSE}_{it}$ is the secondary school enrollment rate; and ${\epsilon }_{it}$ represents the error term.

 [52] show that accurate measurement of social indicators such as under-five and maternal mortality is notoriously difficult in developing countries. The ordinary least squares (OLS) estimator fitted for panel data is valid only under the dual conditions of non autocorrelation of errors and homoscedasticity. When these assumptions are violated, OLS estimators are unbiased but not with minimum variance.

There are a number of estimators that allow us to account for the endogeneity problem (measurement errors and double causality). These are corrected least squares for dummy variables (LSDVC), two-step least squares (2SLS), generalized method of moments (GMM), generalized least squares (GLS), and panel-corrected standard error (PSCE). Each of these methods has its strengths and weaknesses. The last two methods (PSCE and GLS) allow correction for heteroskedasticity and autocorrelation. However, these methods do not account for double causality between an explained variable and an explanatory variable.

The other three methods account for endogeneity but differ from each other. Panel-adjusted least squares has three main advantages over 2SLS and GMM. First, it minimizes bias due to heterogeneity but also due to endogeneity associated with fixed effects. Second, this technique provides good quality estimators for small sample sizes compared to 2SLS and GMM. Finally, LSDV provides unbiased and efficient estimators for systems of equations, assuming that the disturbances between the different equations are uncorrelated.

One of the major weaknesses of the LSDV method is related to the nature of endogeneity. When endogeneity is associated with reverse causality between a dependent and an independent variable, corrected least squares for dummy variables cannot correct for it. While the 2SLS and GMM methods can correct for this problem, they also have limitations. The main difficulty with instrumental variable estimation methods such as double least squares is the choice of instruments.

The generalized moment method (GMM) is appropriate for a dynamic panel. Moreover, for robustness and estimation efficiency, the GMM system of [53] requires that the number of individuals be larger than the number of periods and GMM can provide nonconvergent estimators for panels with reduced individual dimensions. In this study, following the work of [54], we use internal instruments (lagged public health expenditure and GDP variables) and an external instrument (GDP deflator). To check the validity of the instruments, the chi2 tests of [55, 56] are used.

The advantages of the 2SLS method compared with LSDV in this article can be summed up in three points.

First, LSDV gives more robust results within the framework of a system of equations than 2SLS. However, in the context of this article, we have a single linear equation to regress, hence the use of 2SLS.

Secondly, the LSDV method is an appropriate estimation technique for small sample dynamic panel data where GMM cannot be applied efficiently. 2SLS is an approach specifically dedicated to long and continuous series and to static panel data, which is the case of this article.

Finally, the LSDV minimizes the bias linked to the heterogeneity of the panel, although our panel is a homogeneous panel, so 2SLS method is appropriated. **Results**.

### Descriptive statistics

The variables used in the current study and their descriptive statistics are presented in Table 1. The findings reveal that maternal mortality’s maximum and minimum values are around 892 and 62 respectively, for the sample countries. The sampled countries’ public health expenditure had a mean value of about 2.205%, with a standard deviation of 0.652%, a maximum of 4.092%, and a minimum value of 0.906%. The mean value of Gross domestic product per capita was 776,044 USD; the mean value of Incidence of HIV was 2.017, the mean value for population density was nearly 55.19 and its standard deviation was about 34.49. Private health expenditure and Gross secondary school enrolment rate had a mean value of 3.345 and 30.092, with a standard deviation of 0.911 and 15.445, respectively.

**Table 1:** Summary statistics

Variables	Observ	Mean	Std. Dev	Minimum	Maximum
Public health expenditure	161	2.205	0.652	0.906	4.092
Private health expenditure	161	3.345	0.911	2.058	5.444
Gross domestic product per capita	161	776.044	357.312	322.778	1692.545
Population density	161	55.196	34.490	7.757	145.047
Incidence of HIV	161	2.017	1.245	0.1	6.69
Gross secondary school enrolment rate Maternal Mortality Ratio	161 161	30.092 62.410	15.445 143.41	6.391 300	62.002 892

Source: Constructed by authors from World Bank data (2019)

### Multicollinearity test

A high correlation between explanatory variables entails a risk of multicollinearity, making econometric estimates difficult. As such, a correlation matrix is established.

Analysis of Table 2 shows that there is no multicollinearity between the explanatory variables selected in our research. Multicollinearity exists when there are at least two explanatory variables whose correlation coefficients are greater than 0.8 [57]. The study of correlation matrice certainly does not allow us to detect all multicollinearity problems. The most traditional approach is to examine the variance inflation factor (VIF). VIFs estimate how much the variance of a coefficient is increased due to a linear relationship with other predictors. The results of the correlation matrix are confirmed by calculating the VIF and its inverse 1/VIF (Table 2). The VIF coefficients are less than 5, indicating an absence of multicollinearity.

**Table 2:** Correlation Matrix and variance inflation factor

variables	PHE	PrHE	SSE	PDs	IncVIH	GDP
PHE	1					
PrHE	-0.318	1				
SSE	-0.129	-0.256	1			
PDs	-0.178	-0.466	0.731	1		
INCVIH	-0.471	0.2499	0.001	0.207	1	
GDP	-0.244	-0.058	0.345	0.262	0.182	1
**VIF**	1.51	1.84	2.63	3.48	1.57	1.22
**Tolerance**	0.661	0.542	0.380	0.287	0.638	0.820

Source: Constructed by authors from World Bank data (2019)

### Estimation results

Table 3 below presents the overall results of the relationship between public health expenditure and maternal mortality in WAEMU. In general, we have a good goodness of fit in terms of the goodness of fit coefficient (R2) and the associated chi2 probability. Moreover, the overidentification test of Sargan and Basmann for the 2SLS method confirms the validity of the instruments. Finally, the estimation results obtained with the two methods (PCSE and 2SLS) are almost identical. Thus, overall, the estimation results are stable.

**Table 3:** Estimated effect of public health expenditure on maternal mortality ratio per PCSE and 2SLS

Variables	PSCE Estimator	2SLS Estimator
	Maternal mortality	Maternal mortality
**Public health expenditure**	**-0.007**	-0.078***
	**(0.009)**	**(0.029)**
Private health expenditure	-0.003*	0.110***
	(0.002)	(0.015)
Gross domestic product per capita (log)	-0.051	0.019
	(0.051)	(0.022)
Incidence of HIV	0.051***	0.046***
	(0.012)	(0.011)
Gross secondary school enrolment rate	-0.003*	-0.002*
	(0.002)	(0.001)
Population density	-0.004***	-0.004***
	(0.001)	(0.001)
intercept	6.803***	6.181***
	(0.314)	(0.211)
Observations	161	154
R-squared Overall significance test Test of Sargan	0.993 Chi2 = 214.30 Pro > chi2 = 0.00	0.777 Chi2 = 628.24 Pro > chi2 = 0.00 Chi2 = 3.919 (*P* = 0.140)

Statistical notes: standard deviations are in parentheses; significance levels are defined as follows: *** significance at 1%, ** significance at 5%, * significance at 10%

Source: Constructed by authors from World Bank data (2019)

The results show that an increase in public health expenditure of 1% point leads to a reduction in maternal mortality of about 7.8%. Similarly, gross secondary school enrolment rate and population density lead to an improvement in maternal health respectively of about 0.2% and 0.4%. Unlike, the results show that increasing the level of incidence of HIV and private health expenditure by 1% point lead to an increase in maternal mortality of about 4.6% and 11% respectively. However, gross domestic product per capita was non-significant predictor of maternal mortality, hence he does not make contribution to the prediction of maternal mortality ratio.

## Discussion

Higher public spending means better health care for pregnant women, which contributes to better health during pregnancy and postpartum and consequently reduces the mortality rate in this social class. This result is all the more justified given that women are one of the most marginalized social classes in terms of access to health care. Thus, if the government allocates more resources specifically for women, it would reduce the overall financial bottlenecks, especially those of women. This result is consistent with the findings of [20, 23, 58].

Private health expenditure is positively and significantly related at the 1% level to the maternal mortality ratio. This result could be explained by the fact that private healthcare spending limits access to health services for the population in general. Since health is not a pure public good, healthcare consumption is influenced by both public and private spending. In countries where the majority of the population lives below the poverty line, an increase in private spending reduces access to healthcare services through out-of-pocket payments. In the WAEMU, direct household payments account for a significant portion of private healthcare expenditure. This limits access to health care and causes the poor to postpone medical checkups [25, 59]. This finding is consistent with the findings of [60] for developing countries.

The incidence of HIV/AIDS is positively and significantly related to the maternal mortality rate at a threshold of 1%. HIV is a virus that severely weakens the immune system through the loss of antibodies; this can promote the onset of various diseases. This could lead to an increase in the maternal mortality ratio. This result is consistent with the findings of [6] for Sub-Saharan African countries and [29] for Nigeria.

We also find that increasing in gross secondary school enrolment rate is associated with low maternal mortality rate. Education encourages demand for maternal healthcare through several channels. Indeed, education enables people to earn an income through access to employment and to acquire information and knowledge that can change or strengthen health behaviour. Finally, education in general, and education for women in particular, leads to autonomy in decision-making.

Population density is positively and significantly related to the maternal mortality ratio. High population concentration can lead to a reduction in the unit cost of health care. Thus, the government could invest in health care when there is a high population concentration. Additionally, in terms of health infrastructure standards, population density, or at least the number of people in a given area, is a key variable in the provision of such infrastructure. According to the WHO, the standards for health infrastructure are one hospital, one health center, and one health post per 150,000 inhabitants, 50,000 inhabitants, and 10,000 inhabitants, respectively. In addition, population density can be a source of income for those who make investments. This contributes to an increase in their income and consequently facilitates access to health centers. This result is consistent with the findings of [49].

### Strengths and limitations of the study

A limitation of this study was a high rate of missing data for some variables, such as the ratio of physicians to the population, which resulted in these variables being excluded from the study. The use of the proxy data is also a limitation of the paper as the use of secondary school enrolment to proxy for the proportion of female adult population with secondary school education.

Despite this limitation, this study is the first of its kind to examine the relationship between public health expenditure and maternal mortality in WAEMU.

## Conclusion

The aim of this article was to analyze the direct effect of public health expenditure on the maternal mortality ratio in the WAEMU. The PCSE and 2SLS methods were used. Overall, the results are consistent with economic theory. In general, authorities need to increase public health spending to build more health centers and improve the equipment of existing infrastructures. In particular, an improved version of free health care for pregnant women in Burkina Faso, Niger, Senegal and Benin should be extended to the other WAEMU countries. In addition to applying this policy in other countries, rigorous monitoring and evaluation are needed to correct any shortcomings. This could be achieved by implementing the financing mechanism, ensuring regular availability of medicines and supplies at health centers, and increasing information to women (especially pregnant women) about free health care.

Private spending on healthcare is a barrier to improving maternal health in WAEMU. When the population lacks sufficient income, an increase in public spending is not necessarily accompanied by an improvement in health outcomes. Policies aimed at reducing household copayments could reduce financial barriers to accessing healthcare and increase the consumption of healthcare services, especially in WAEMU countries, where the majority of households are poor. Therefore, it is essential to accelerate the introduction of universal health insurance or household prepayment schemes and new approaches to effectively improve the level of education in order to achieve a more rapid reduction in maternal mortality.

Authorities could also encourage policies aimed at reducing the incidence of HIV/AIDS, promoting awareness-raising among young people and education on reproductive health.

## Data Availability

All the data used in the empirical analysis are publically available and can be downloaded from the following website: https://databank.worldbank.org/source/world-development-indicators.
